# Everolimus- and sirolimus-eluting stents in patients with and without ST-segment elevation myocardial infarction

**DOI:** 10.1007/s12471-014-0525-0

**Published:** 2014-02-13

**Authors:** M. A. Velders, A. J. van Boven, J. Brouwer, P. C. Smits, A. W. J. van ’t Hof, C. J. de Vries, M. Queré, S. H. Hofma

**Affiliations:** 1 Department of Cardiology, Medical Center Leeuwarden, PO Box 888, 8901 BR Leeuwarden, the Netherlands; 2 Department of Cardiology, Maasstad Hospital, Rotterdam, the Netherlands; 3 Department of Cardiology, Isala Clinics, Zwolle, the Netherlands

**Keywords:** Drug-eluting stents, Percutaneous coronary intervention, Coronary artery disease

## Abstract

**Aims:**

Everolimus-eluting stents (EES) were superior to sirolimus-eluting stents (SES) in a dedicated myocardial infarction trial, a finding that was not observed in trials with low percentages of ST-elevation myocardial infarction (STEMI). Therefore, this study sought to investigate the influence of clinical presentation on outcome after EES and SES implantation.

**Methods:**

A pooled population of 1602 randomised patients was formed from XAMI (acute MI trial) and APPENDIX-AMI (all-comer trial). Primary outcome was cardiac mortality, MI and target vessel revascularisation at 2 years. Secondary endpoints included definite/probable stent thrombosis (ST). Adjustment was done using Cox regression.

**Results:**

In total, 902 EES and 700 SES patients were included, of which 44 % STEMI patients (EES 455; SES 257) and 56 % without STEMI (EES 447; SES 443). In the pooled population, EES and SES showed similar outcomes during follow-up. Moreover, no differences in the endpoints were observed after stratification according to presentation. Although a trend toward reduced early definite/probable ST was observed in EES compared with SES in STEMI patients, long-term ST rates were low and comparable.

**Conclusions:**

EES and SES showed a similar outcome during 2-year follow-up, regardless of clinical presentation. Long-term safety was excellent for both devices, despite wide inclusion criteria and a large sub-population of STEMI patients.

## Introduction

Drug-eluting stents (DES) were designed to reduce the in-stent neointimal hyperplasia that commonly occurred in bare-metal stents (BMS). Indeed, first-generation DES (i.e. paclixatel-eluting stents (PES) and sirolimus-eluting stents (SES)) reduced the need for revascularisation procedures compared with BMS but were associated with higher rates of late stent thrombosis (ST), especially in complex patients such as those presenting with myocardial infarction (MI) [1–3].

Delayed arterial healing and stent malapposition were found to play a role in the higher ST rates after DES implantation in the setting of MI [4, 5]. Second-generation DES were designed to be safer and more effective through changes in stent design, polymer and anti-restenotic drugs. So far, second-generation everolimus-eluting stents (EES) have shown superior results to PES in a wide range of indications [6]. Compared with SES, EES have mostly shown comparable outcomes but improvements in ST rates have been observed [7–10]. In contrast, one dedicated trial of predominantly ST-segment elevation myocardial infarction (STEMI) patients showed superiority of EES over SES during short-term follow-up [11]. Long-term randomised data are scarce, especially in setting of STEMI.

This study sought to investigate the influence of clinical presentation on outcome of EES and SES during 2-year follow-up.

## Methods

Patient-level data from the randomised XAMI and APPENDIX-AMI trials were pooled to form the patient population. The design and results of these trials have been published previously [11, 12]. In short, XAMI (NTR1123, http://www.trialregister.nl/trialreg/admin/ctview.asp?TC=1123) was a multicentre, clinical non-inferiority trial randomising 625 acute MI patients to EES (Xience V [Abbott Vascular, Santa Clara, California]) or SES (Cypher [Cordis, Bridgewater, New Jersey]) in a 2:1 ratio. To be enrolled, patients had to have STEMI or non-STEMI with an emergency indication for percutaneous coronary intervention (PCI). Exclusion criteria were: chronic total occlusion as target lesion; known allergy to sirolimus, everolimus, aspirin or clopidogrel; inability to obtain informed consent; life expectancy <1 year; or stent size required to treat lesion >3.5 mm.

APPENDIX-AMI (NTR3170, http://www.trialregister.nl/trialreg/admin/rctview.asp?TC=3170) was a single-centre open-label trial randomising 977 all-comer patients to EES and SES (ratio 1:1). The trial included all patients eligible for coronary revascularisation by PCI for any indication. Exclusion criteria were: known allergy for everolimus or sirolimus, aspirin or clopidogrel; inability or unwillingness to give informed consent; and anatomy in which stent implantation is deemed not technically possible.

Patients were pretreated with loading doses of aspirin and clopidogrel, in addition to intravenous heparin bolus of 5000 IE in case of MI. Use of glycoprotein IIb/IIIa inhibitors, thrombus aspiration and balloon pre-dilatation were left up to the discretion of the operator. Aspirin was recommended for life and clopidogrel for a minimum of 1 year. Protocol-defined follow-up was performed after 30 days, 1 year and 2 years by questionnaires and telephone contact. Follow-up was gathered by research nurses in a blinded fashion. Event adjudication was performed by a blinded clinical event committee in XAMI. In APPENDIX-AMI, event adjudication was performed between physicians on a consensus basis in an unblinded fashion. The study protocols were approved by the local ethics committees of the participating centres and the trials were conducted according to the principles of the Declaration of Helsinki. All patients gave oral consent before enrolment and written informed consent after procedure.

## Definitions

The primary endpoint was a composite of cardiac death, MI and target vessel revascularisation (TVR). MI was defined as a rise of creatine kinase (CK) more than three times the upper limit of normal along with a rise in CK-MB with recurrent symptoms and/or new electrocardiographic changes. In acute coronary syndrome patients, re-infarction within 48 h after index procedure was defined as a re-elevation of CK of >1.5 times the previous value with elevation of CK-MB, along with recurrent symptoms and/or new electrocardiographic changes. MI around coronary artery bypass grafting required a CK rise of >5 times the upper limit of normal. TVR was defined as any repeat percutaneous or surgical intervention on any segment of the target vessel. Other secondary endpoints included the individual components of the composite endpoint, target lesion revascularisation (TLR) and definite or probable ST. TLR was defined as any repeat intervention or bypass grafting of the target lesion previously treated with stenting along with the 5 mm proximal or distal vessel. ST was defined in accordance with the Academic Research Consortium definitions [13].

## Statistical analyses

Comparisons were made according to randomised treatment and presentation with or without STEMI. Continuous variables are presented as means with standard deviations or medians with interquartile range (IQR) and were compared using Student’s *t*-test. Categorical variables are expressed as counts and percentages and were compared by means of Pearson’s *χ* ^2^ test. All statistical tests were 2-tailed and a *p*-value <0.05 was considered statistically significant. Time-to-event analyses were performed using Kaplan-Meier curves, which were compared using log-rank tests. To adjust for unbalanced baseline characteristics, Cox proportional hazards analyses were performed. The proportional hazards assumption was investigated visually. Adjusted effect sizes were calculated for primary and secondary endpoints with a p-value less than 0.10 as judged by log rank test. Adjustment was performed for characteristics significantly differing between groups (*p* < 0.05), which were incorporated in the multivariable models. Analyses were repeated with a variable stating the trial the patient originated from, to evaluate the influence of individual trials on the results.

To avoid dropping of events due to missing baseline information, multiple imputation was performed for the baseline variables that were included in the multivariable models: presence of heavy calcification was unknown in four patients (three EES and one SES) and total stent length was unknown in four patients (three EES and one SES). Reasons for missing data were unknown and assumed to be random. Total stent length was log transformed to meet the assumption for normal distribution. Missing data values were imputed for heavy calcification and total stent length using the following predictors: age, gender, cardiac risk factors, cardiac history, renal insufficiency, indication for PCI, target lesion, lesion type, number of vessel disease, heavy calcification, total stent length, max stent diameter, and number of stents per patient. Twenty imputed datasets were created and Cox proportional hazards analyses were performed on the pooled datasets [14]. Analyses were performed using IBM SPSS version 21.

## Results

In total, 1602 patients were randomised in the XAMI and APPENDIX-AMI trials, of which 902 to EES and 700 to SES. Two-year follow-up data were available for 1575 patients (98.3 %). The presenting diagnosis was stable angina in 526 patients (32.8 %), unstable angina or non-STEMI in 364 patients (22.7 %) and STEMI in 712 patients (44.4 %). After pooling of the two trials, the primary endpoint occurred in 8.8 % of EES patients vs. 10.2 % of SES patients during 2-year follow-up in the overall population, HR 0.86 (95 % CI 0.62–1.18), *p* = 0.347. Secondary endpoints were also balanced between the groups.

## Stratification on presenting diagnosis

STEMI patients were younger than patients without STEMI and more likely to smoke but had lower rates of comorbidity and other risk factors (Table 1). Coronary thrombus was more common in STEMI, but rates of heavy calcification, bifurcations and multivessel disease were lower. Stent length and number of stents used were also lower in STEMI, but stent diameter was slightly larger. Finally, glycoprotein IIb/IIIa inhibitor use was more common in STEMI while TIMI 3 flow after procedure was less often achieved. During 2-year follow-up, STEMI patients showed lower rates of the primary endpoint (7.2 % vs. 11.2 %, *p* = 0.007) and TLR (1.4 % vs. 4.1 %, *p* = 0.001) compared with patients without STEMI.

**Table 1:** Baseline characteristics

	STEMI			Other indications		
Variable	EES (*N* = 455)	SES (*N* = 257)	*p* value	EES (*N* = 447)	SES (*N* = 443)	*p* value
Age, years	61.8 ± 11.4	62.4 ± 11.5	0.501	65.2 ± 11.3	65.1 ± 11.1	0.868
Male	329 (72.3 %)	194 (75.5 %)	0.356	319 (71.4 %)	319 (72.0 %)	0.831
Diabetes mellitus	41 (9.1 %)	26 (10.2 %)	0.612	73 (16.9 %)	80 (18.6 %)	0.492
Hypertension^a^	136 (30.1 %)	81 (31.8 %)	0.643	191(43.6 %)	223 (51.5 %)	0.020
Hypercholesterolaemia^b^	124 (27.8 %)	60 (23.8 %)	0.250	242 (57.5 %)	246 (57.9 %)	0.906
Current smoker	232 (51.3 %)	135 (53.1 %)	0.642	121 (27.6 %)	94 (21.9 %)	0.051
Prior myocardial infarction	32 (7.0 %)	19(7.4 %)	0.858	100 (22.6 %)	102 (23.2 %)	0.844
Prior PCI	19 (4.2 %)	9 (3.5 %)	0.653	81 (18.2 %)	105 (23.7 %)	0.046
Prior CABG	4 (0.9 %)	5 (1.9 %)	0.221	47 (10.5 %)	71 (16.0 %)	0.016
Prior renal insufficiency	8 (1.8 %)	6 (2.4 %)	0.596	49 (11.8 %)	43 (10.6 %)	0.598
Presenting diagnosis						0.072
- Stable angina	0 (0.0 %)	0 (0.0 %)	–	251 (56.2 %)	275 (62.1 %)	
- Unstable angina or non-STEMI	0 (0.0 %)	0 (0.0 %)	–	196 (43.8 %)	168 (37.9 %)	
- STEMI	455 (100 %)	257(100 %)	–	0 (0.0 %)	0 (0.0 %)	–
Symptoms to first medical contact (min)	90 (60–170)	100 (60–185)	0.419	–	–	
First medical contact to balloon inflation (min)	75 (60–100)	75 (60–100)	0.937	–	–	

Data are expressed as mean ± SD, as number (percentage), or as median (interquartile range). CABG = coronary artery bypass grafting

^a^Blood pressure 140/90 mm Hg or previous pharmacological treatment

^b^Total cholesterol 190 mg/dl or previous pharmacological treatment

**Fig. 1:**
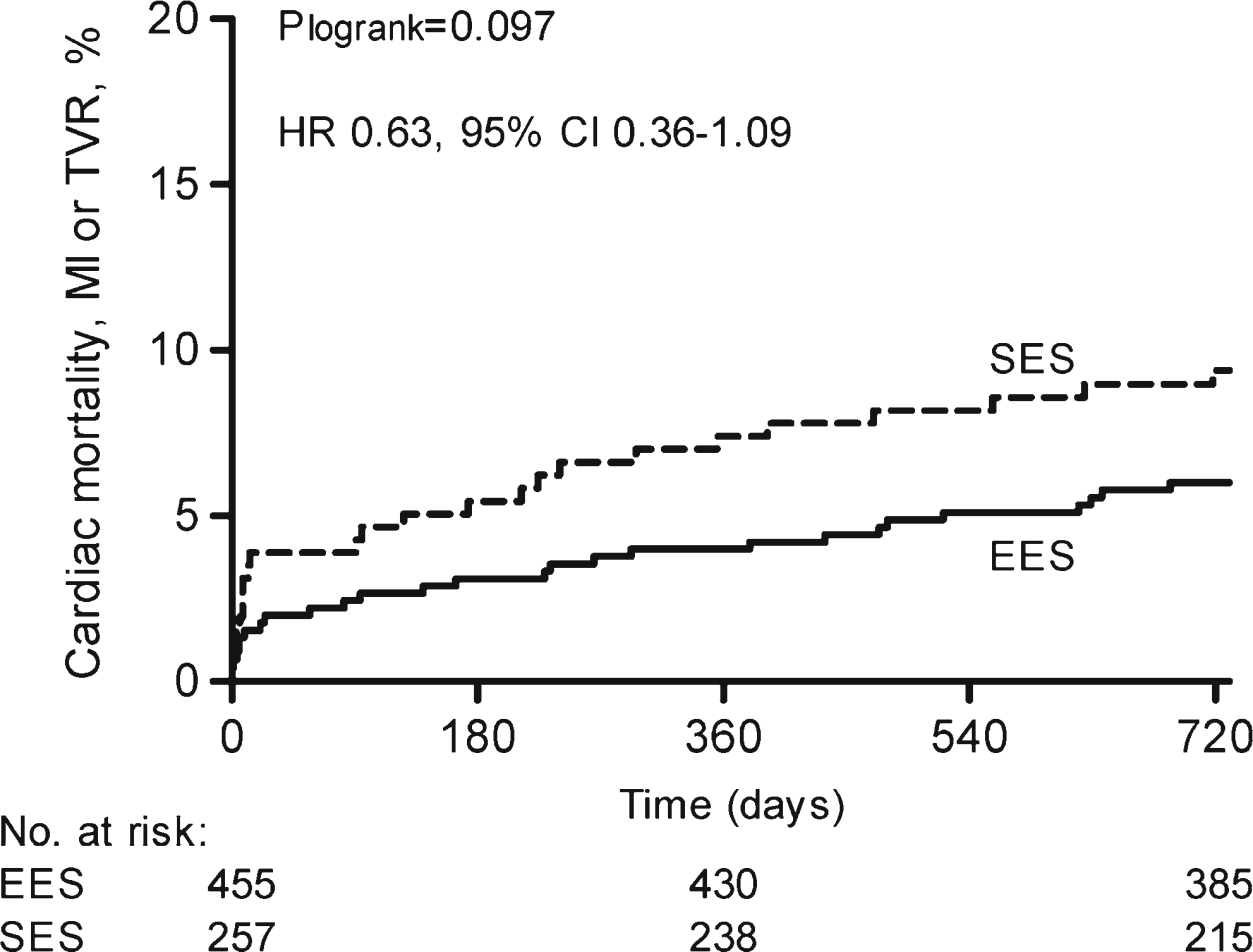
Two-year primary outcome according to randomised stent in STEMI patients

**Fig. 2:**
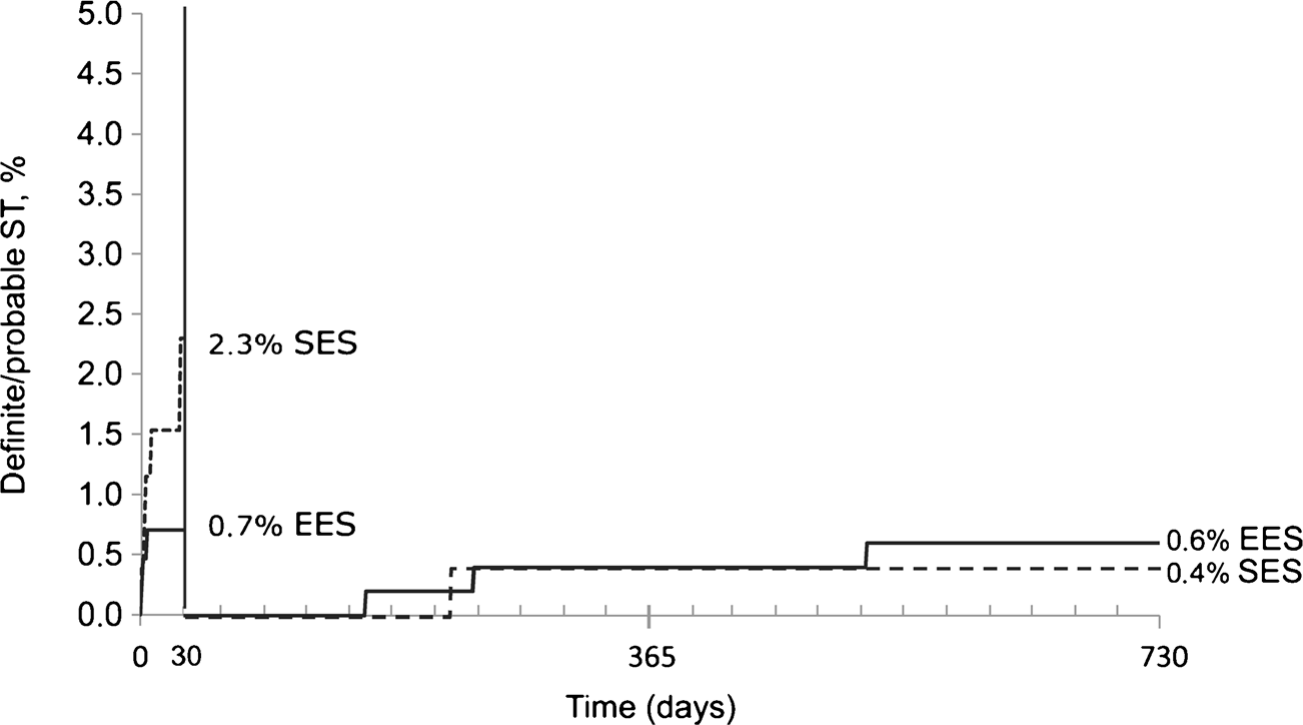
Definite/probable stent thrombosis with landmark analysis at 30-days in STEMI population

In the STEMI population, EES showed less calcified lesions and total stent length was shorter compared with SES (Table 2). During 2-year follow-up, randomisation to EES resulted in a similar primary endpoint rate (unadjusted HR 0.63, 95 % CI 0.36–1.09, *p* = 0.097, adjusted HR 0.66, 95 % CI 0.38–1.15, *p* = 0.141) compared with SES (Table 3, Fig. 1). A trend was observed for reduced early definite/probable ST in EES. However, long-term ST rates were low and similar (Fig. 2). At 1-year, aspirin (or coumadin) compliance was 94.8 % in EES versus 91.5 % in SES (p = 0.092). Thienopyridine compliance was 95.6 % in EES versus 91.8 % in SES (p = 0.040). Two patients were not on dual antiplatelet therapy at the time of ST: 1 EES and 1 SES patient, both suffering probable ST.

**Table 2:** Procedural characteristics

	STEMI			Other indications		
Variable	EES (*N* = 455)	SES (*N* = 257)	*p* value	EES (*N* = 447)	SES (*N* = 443)	*p* value
Target coronary lesion			0.651			0.108
- Left main artery	0 (0.0 %)	1 (0.4 %)		26 (5.9 %)	15 (3.4 %)	
- Left anterior descending artery	175 (38.5 %)	104 (40.5 %)		195 (44.0 %)	175 (39.8 %)	
- Left circumflex artery	86 (18.9 %)	50 (19.5 %)		96 (21.7 %)	124 (28.2 %)	
- Right coronary artery	192 (42.3 %)	101 (39.3 %)		124 (28.0 %)	124 (28.2 %)	
- Bypass graft	1 (0.2 %)	1 (0.4 %)		2 (0.5 %)	2 (0.5 %)	
Multivessel disease	206 (45.3 %)	130 (50.6 %)	0.173	249 (55.8 %)	252 (56.9 %)	0.751
Bifurcation intervention	55 (12.1 %)	37 (14.4 %)	0.390	107 (24.4 %)	98 (22.4 %)	0.496
Heavy calcification	26 (5.8 %)	28 (10.9 %)	0.013	62 (14.0 %)	85 (19.4 %)	0.032
Lesion type B2/C	300 (66.7 %)	171 (67.6 %)	0.803	245 (55.4 %)	241 (55.0 %)	0.903
Visible thrombus	383 (84.5 %)	223 (87.1 %)	0.352	49 (11.1 %)	39 (8.9 %)	0.276
Thrombosuction	250 (54.9 %)	142 (55.3 %)	0.937	11 (2.5 %)	6 (1.4 %)	0.222
Total stent length (mm)	25.3 ± 14.7	27.7 ± 16.5	0.046	28.2 ±18.9	28.2 ±16.0	0.986
Max stent diameter (mm)	3.1 ± 0.4	3.1 ± 0.3	0.676	3.1 ± 0.6	3.1 ± 0.5	0.436
No. of stents/patients	1.4 ± 0.7	1.4 ± 0.7	0.396	1.5 ± 0.7	1.5 ± 0.8	0.475
Glycoprotein IIb/IIIa inhibitor treatment	346 (76.0 %)	196 (76.3 %)	0.947	98 (22.3 %)	82 (18.7 %)	0.181
Postprocedural TIMI flow grade 3	431 (94.9 %)	238 (92.6 %)	0.206	428 (98.2 %)	420 (97.0 %)	0.262

Data are expressed as number (percentage) or as mean ± SD

*TIMI* thrombolysis in myocardial infarction

**Table 3:** Clinical endpoints at 2 years

	STEMI			Other indications		
Variable	EES (*N* = 450)	SES (*N* = 257)	*p* value	EES (*N* = 436)	SES (*N* = 432)	*p* value
Primary composite endpoint^a^	27 (6.0)	24 (9.3)	0.099	51 (11.7)	46 (10.6)	0.624
Mortality						
- All-cause	15 (3.3)	14 (5.4)	0.173	25 (5.7)	19 (4.4)	0.370
- Cardiac	10 (2.2)	8 (3.1)	0.470	16 (3.7)	10 (2.3)	0.242
Myocardial infarction	6 (1.3)	5 (1.9)	0.527	6 (1.4)	8 (1.9)	0.578
Target vessel revascularisation	15 (3.3)	13 (5.1)	0.258	33 (7.6)	32 (7.4)	0.928
Target lesion revascularisation	7 (1.6)	3 (1.2)	0.674	19 (4.4)	17 (3.9)	0.755
Stent thrombosis						
- Definite	3 (0.7)	0 (0.0)	0.190	3 (0.7)	4 (0.9)	0.695
- Definite/probable	6 (1.3)	7 (2.7)	0.186	4 (0.9)	5 (1.2)	0.727
- Early	3 (0.7)	6 (2.3)	0.057	1 (0.2)	2 (0.5)	0.558
- Late	2 (0.4)	1 (0.4)	0.913	0 (0.0)	1 (0.2)	0.315
- Very late	1 (0.2)	0 (0.0)	0.449	3 (0.7)	2 (0.5)	0.661

Values are expressed as number (percentage)

^a^Cardiac mortality, myocardial infarction and target vessel revascularisation

**Fig. 3:**
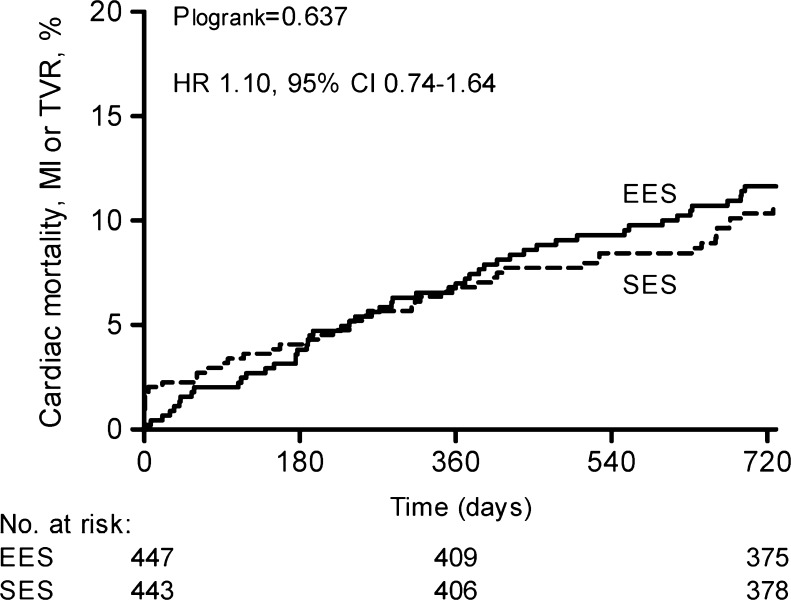
Two-year primary outcome according to randomised stent in patients without STEMI

**Fig. 4:**
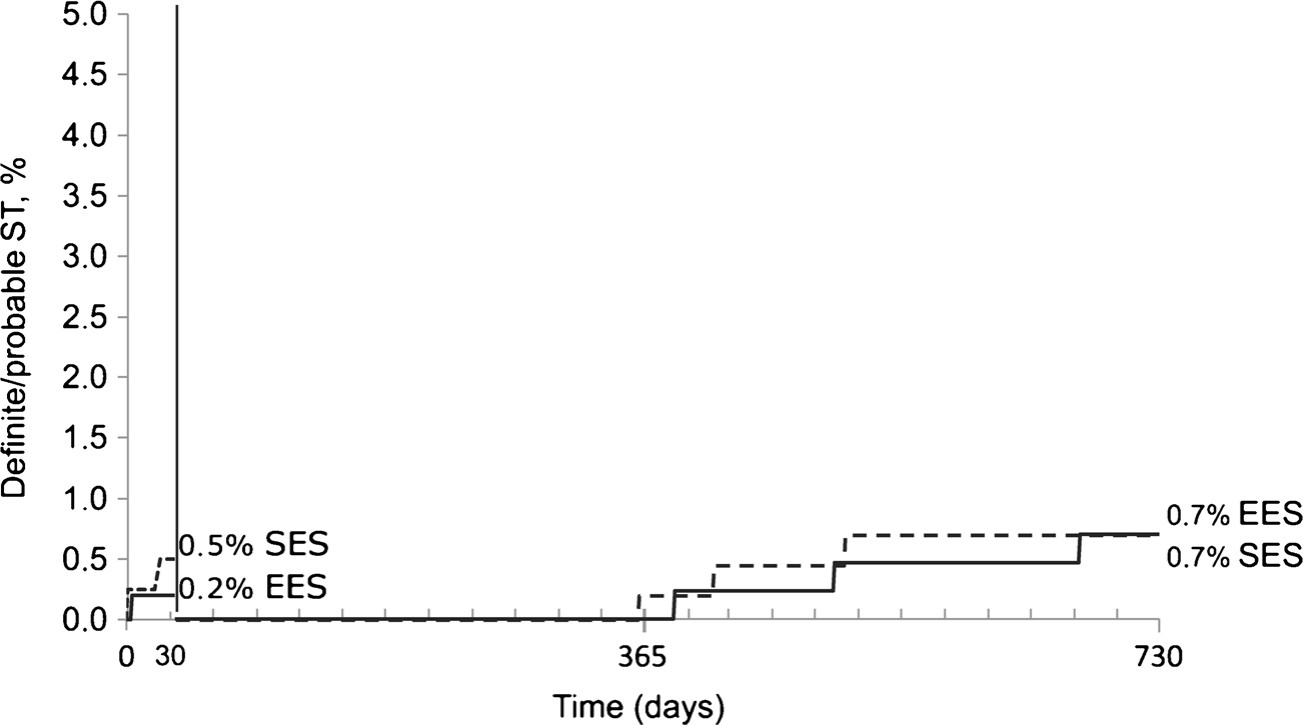
Definite/probable stent thrombosis with landmark analysis at 30-days in population without STEMI

In the population without STEMI, EES patients showed lower rates of hypertension, prior PCI, bypass grafting and heavy calcification compared with SES patients (Tables 1 and 2). At 2 years, EES and SES showed similar rates of the primary endpoint (HR 1.10, 95 % CI 0.74–1.64, *p* = 0.637) (Table 3, Fig. 3). Other secondary endpoints were also balanced. Definite/probable ST rates were low and similar between the groups (Fig. 4). Aspirin compliance during 1 year was 97.6 % in EES and 99.3 % in SES (*p* = 0.047). Thienopyridine compliance was 96.8 % in EES and 97.5 % in SES (*p* = 0.518).

The *p*-value for interaction between randomised stent and presenting diagnosis (STEMI vs. other) was 0.104 (HR 1.76, 95 % CI 0.89–3.46) for the primary endpoint.

## Discussion

The present pooled analysis of the randomised XAMI and APPENDIX-AMI trials provided 2-year outcome data of EES and SES according to clinical presentation. The performance of the first- and second-generation DES was found to be similar and independent of clinical presentation. Importantly, despite wide inclusion criteria and a large sub-population of STEMI patients, both devices showed a comparable safety at long-term follow-up.

Implantation of first-generation DES in the previously off-label indication of acute MI was controversial until publication of the HORIZONS-AMI trial, which confirmed the safety and efficacy of PES compared with BMS in primary PCI [15]. Superiority of second-generation EES over PES in acute coronary syndromes has been established but data comparing EES with previous golden standard SES are less abundant, especially in setting of STEMI [16]. In SORT OUT IV, EES and SES showed comparable outcomes up to 2 years, with the exception of a lower rate of definite ST in EES patients [7]. However, only 10 % of patients presented with STEMI. The other major trials that compared EES and SES showed no differences in outcome in up to 3 years of follow-up [8–10]. Also in these trials, the STEMI population was strongly underrepresented, making randomised data of EES and SES in STEMI patients scarce beyond 1 year. In contrast, almost half the patients included in the current study presented with STEMI.

In the STEMI population of the present study, event rates were lower than in the population without STEMI, likely explained by the generally less complex thrombotic lesions in STEMI. Although EES appeared to perform slightly better than SES, no significant differences in the primary outcome measure were observed. Nonetheless, a strong trend toward reduced early definite/probable ST hinted at a possible advantage of EES over SES in the early phase after MI. In contrast, clinical outcomes were balanced between EES and SES in patients presenting with a diagnosis other than STEMI, which is in accordance with previous trials [8–10].

Findings observed in the STEMI population were comparable with reports of the EXAMINATIONS trial, in which EES use resulted in a lower rate of early definite/probable ST compared with BMS in STEMI patients [17]. At 1-year, the definite/probable ST rate was 0.9 % in the EES group, comparable with the 1.1 % rate observed in this study. Additionally, Kalesan et al. performed a propensity matched comparison of EES and SES in ACS patients and found a reduction in both the primary endpoint and ST during 3-year follow-up [18].

Important differences of EES compared with SES are the thin strut design and the biocompatible polymer. In ex-vivo and in-vivo models, thin struts were less thrombogenic and the slim design of EES has been associated with faster endothelialisation compared with SES [19, 20]. Also, the biocompatible polymer of EES may be associated with a reduced long-term inflammatory response. While the current study found reassuringly low rates of ST in both DES, very long-term monitoring is necessary to establish a potential benefit of EES over SES. In the TYPHOON trial, SES showed a late ‘catch-up’ phenomenon for ST, i.e. the relatively low early ST rates were abolished by higher very late ST rates compared with BMS in STEMI patients during 4 years of follow-up [21].

Although the polymer applied in EES is more biocompatible than the SES polymer coating, additional improvement may be achieved with a biodegradable polymer coating. The COMFORTABLE AMI trial compared biolimus-eluting stents with BMS in STEMI patients but did not find a reduction in 1-year definite/probable ST [22]. However, long-term follow-up will have to show if STEMI patients benefit from DES with biodegradable polymer, as the main effect of a biodegradable polymer in reducing ST becomes evident after 1 year [23].

## Limitations

Our study is limited by its post hoc nature and therefore findings should be considered hypothesis generating. XAMI included only patients with acute MI, while APPENDIX-AMI included all-comer patients. Furthermore, the randomisation rate differed between XAMI (2:1) and APPENDIX-AMI (1:1) which created baseline misbalance between the groups, although multivariable corrections were performed to adjust for these differences. Finally, the analysis was underpowered to detect differences in the ST rates.

## Conclusions

The present pooled analysis of the XAMI and APPENDIX-AMI trials found similar outcomes between EES and SES during 2 years of follow-up, regardless of clinical presentation. Long-term ST rates were reassuringly low in both stent types.
